# Tonal Tinnitus Does Not Interfere with Tone Detection at the Tinnitus Pitch-Matched Frequency

**DOI:** 10.1177/23312165251376382

**Published:** 2025-09-08

**Authors:** J Gerard G Borst, André Goedegebure

**Affiliations:** 1 Department of Neuroscience, Erasmus MC, 6993 University Medical Center Rotterdam, Rotterdam, the Netherlands; 2 Department of Otolaryngology, Erasmus MC, 6993 University Medical Center Rotterdam, Rotterdam, the Netherlands

**Keywords:** tinnitus, psychometric function, hearing threshold, pitch

## Abstract

Individuals with tinnitus hear sounds that are not present in the external environment. Whereas hearing difficulties at frequencies near those matching the tinnitus pitch are a common complaint for individuals with tinnitus, it is unclear to what extent the internal tinnitus sounds interfere with the detection of external sounds. We therefore studied whether pure-tone detection at the estimated frequency corresponding to the tinnitus pitch (f_tp_) was affected by confusion with the tinnitus percept. Signs of confusion would be a high false alarm rate or a shallower slope of the psychometric function for tone detection at f_tp_. We selected participants with symmetric, tonal tinnitus, who were able to estimate its pitch consistently (n = 18). Another 18 participants matched for high-frequency hearing loss, age, and sex, but without tinnitus, served as the control group. For both groups, we measured the psychometric function for detecting long-duration tones, maximizing the likelihood for confusion with an external sound. We observed that false alarm rates for tinnitus participants were not higher for test tones at f_tp_, nor were they higher than for the control group without tinnitus. Similar results were obtained for the slopes of the psychometric functions. Apparently, individuals with tinnitus are well able to discriminate between their own tinnitus and comparable external sounds. Our results indicate that (tonal) tinnitus does not interfere with the detection of soft sounds at the tinnitus pitch-matched frequency.

## Introduction

Individuals with tinnitus hear sounds that are not present in the environment. Tinnitus is remarkably common: it is present in about 1 in 7 adults, and about 1 in 50 adults have a severe form of tinnitus, impairing their quality of life significantly (Jarach et al., 2022). The tinnitus loudness is not strongly related to the distress it causes (Fowler, 1944). The prevalence of both tinnitus and hearing loss increases with age (Biswas et al., 2022; Jarach et al., 2022), although this increase is less evident for tinnitus than for hearing loss (Oosterloo et al., 2021), and it is commonly believed that there is a causal connection, i.e., that tinnitus is initiated by some form of impaired hearing (Baguley et al., 2013; Biswas et al., 2023; Henton & Tzounopoulos, 2021; Shore & Wu, 2019). Individuals with tinnitus may complain that their tinnitus interferes with their hearing; several papers have suggested that tinnitus can interfere with the detection of a pure tone with a frequency near the tinnitus pitch-matched frequency (Douek & Reid, 1968; Fowler, 1944; Josephson, 1931; Minton, 1923), and Jones and Knudsen (1928) wrote: “Of course a tinnitus of any type will produce a masking effect, and this of itself would interfere with normal hearing.” The American Speech-Language-Hearing Association currently recommends use of a pulsed signal or a warble tone for threshold audiometry for individuals with tinnitus to help them distinguish the test signal from their tinnitus (ASLHA, 2005).

How might tinnitus interfere with detection of external sounds? An increase in spontaneous firing of auditory neurons is thought to be one neural correlate of tinnitus (Eggermont & Roberts, 2004). Putative mechanisms underlying this increase in spontaneous firing rate include an increase in central gain (Noreña, 2011; Schaette & McAlpine, 2011) and an increase in central noise (Zeng, 2013). It has been well established that detection of a sensory stimulus that is close to threshold depends on neural activity immediately preceding the presentation of the stimulus, both in audition (Buran et al., 2014; Sadaghiani et al., 2009) and in other sensory modalities (Boly et al., 2007; Karvat et al., 2021; Morton et al., 2024; Wu et al., 2024; Yoshida & Katz, 2011), suggesting that spontaneous firing can affect the detection of near-threshold sensory stimuli. This opens up the possibility that tinnitus interferes with sound detection. At the cellular level, an increase in spontaneous firing in tinnitus might translate to increased activity of the inputs to, for example, neurons in the auditory cortex. Both simulations and experimental work have shown that an increase in spontaneous synaptic activity is expected to lead to multiplicative changes (i.e., slope increases) in the input–output relation which are called “gain modulation” (reviewed in (Haider & McCormick, 2009; Silver, 2010). At the psychophysical level, these increases in the slope of the input–output curve might translate to an increase in the slope of the psychometric function. However, Penner (1986) found that for tinnitus participants the slope of the psychometric function for tone detection was shallower for the tinnitus pitch-matched frequency than for both a lower and a higher frequency. False-positive detections were not evaluated due to the type of task that was employed (two-alternative forced choice; 2-AFC). The more shallow slope was hypothesized to be due to fluctuations in the tinnitus loudness, which may have contributed to uncertainty in the external tone detection (Penner, 1986).

Other findings cast doubt on the idea that tinnitus interferes with auditory detection. A strong relation between increased spontaneous firing and behavioral evidence for tinnitus has not been universally found in animal models of tinnitus (Coomber et al., 2014; Longenecker & Galazyuk, 2016; Ropp et al., 2014). Moreover, the tinnitus percept does not have the same psychophysical characteristics as the percept evoked by external tones (Burns, 1984; Feldmann, 1971; McFadden & Wightman, 1983; Penner, 1987; Penner & Klafter, 1992). For example, whereas masking of an external tone is strongly frequency-dependent, masking of tinnitus is in many cases largely independent of the frequency of the masker, suggesting that the tinnitus percept is fundamentally different from the response to an external sound stimulus (Feldmann, 1971; Fournier et al., 2019; Penner, 1987; Penner & Klafter, 1992). Whereas the observation that tinnitus in many individuals sounds like a tone suggests a highly localized involvement of tonotopically organized neurons, this frequency-independent masking at the same time suggests involvement of neurons with broad tuning, or from areas without a strict tonotopic organization (Fournier et al., 2019). It has been difficult to devise an auditory test that can objectively discriminate between individuals with and without tinnitus when hearing loss was controlled for (Langers et al., 2012; Long et al., 2023; Nieschalk et al., 1998; Omidvar et al., 2018; Ravirose et al., 2019; Reisinger et al., 2024; Tan et al., 2013; Zeng et al., 2020), whereas if tinnitus interferes with hearing, this might be the basis for an objective tinnitus test. In summary, while there is evidence that tinnitus affects sound detection, this evidence is limited, and the impact tinnitus would have on the psychometric function at the tinnitus pitch-matched frequency is uncertain.

Here, we revisited the question of whether tinnitus interferes with sound detection. We tested whether individuals with tinnitus experience difficulties discriminating between silence and sounds that are matched to their tinnitus. More specifically, we tested whether the false alarm rate is increased and the slope of the psychometric function for tone detection is different at the tinnitus-matched frequency compared to 0.6 oct below, and compared to matched control participants. To maximize the probability of confusion, we selected individuals with tonal tinnitus and we used long tones to have a stimulus that resembles tinnitus more than short duration tones.

## Materials and Methods

### Participants

Participants were Dutch speaking adults. Most were recruited via social media. Individuals who underwent psychometric tests as part of the study gave informed written consent for participation. They received only reimbursement of travel costs. The study conformed to the Declaration of Helsinki; it was submitted to the Institutional Ethical Committee under number MEC-2020-0380, who waived the requirement for approval.

### Psychometric Tests

**Figure 1.**
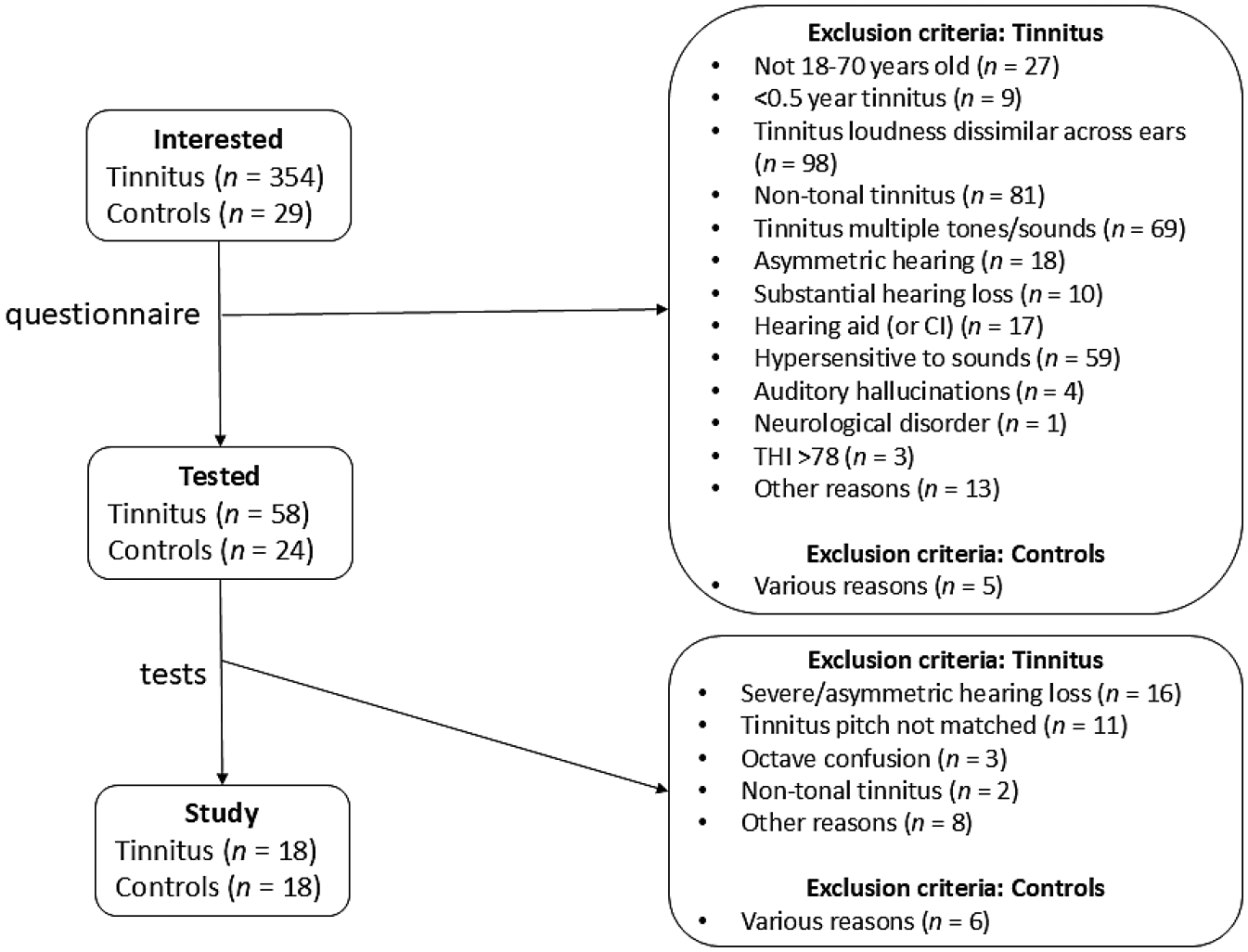
Selection of Participants. People Interested in the Study Were Asked to Fill Out a Questionnaire Assessing the Inclusion and Exclusion Criteria (Table 1) and a Dutch Version of the Tinnitus Handicap Inventory (Newman et al., 1996). The Categories “Other Reasons” or “Various Reasons” Included No Time. In One Case, the Tinnitus Turned Out to Be the Neighbors' Air-Conditioning.

Inclusion and exclusion criteria are summarized in Table 1. Figure 1 summarizes the selection of participants. All tests were performed in a double-walled soundproof room. Tests were performed in the order in which they are described here. For all potential participants, the first test consisted of estimating hearing thresholds with a Decos Audiology Workstation at 0.25, 0.5, 1, 2, 4, and 8 kHz for air conduction and 0.5, 1, 2, and 4 kHz for bone conduction with a minimum step size of 5 dB (ASLHA, 2005). Tones were delivered with a TDH-39P Telephonics headset and a B71 RadioEar bone transducer.

**Table 1.** Inclusion and Exclusion Criteria for the Participants.

Inclusion Criteria	Exclusion Criteria
None to moderate hearing loss (≤45 dB HL average of 0.5, 1, 2, and 4 kHz air conduction for one or both ears)	Asymmetric hearing loss: >10 dB at f_tp_ or 0.6 oct below f_tp_, >20 dB average of 0.5, 1, 2, and 4 kHz air conduction)
Between 18 and 70 years of age	Air bone gap on average >10 dB at 0.5, 1, 2 and 4 kHz or >15 dB for two consecutive frequencies for one or both ears
Tinnitus is tonal, binaural (similar loudness for the two ears)	Hearing loss >70 dB HL at f_tp_ or 0.6 oct below f_tp_
Tinnitus present >6 months	Hypersensitive to sounds
Tinnitus can be masked by external sounds	Auditory hallucinations
Tinnitus pitch can be reproducibly matched to an external sound	Neurological disorder
THI score <78	Participation in other clinical studies

The Participants With Tinnitus Had to Pass All Inclusion Criteria, While for the Controls Only the First Two Inclusion Criteria Applied, Where f_tp_ Was Assigned as Discussed Below. The Presence of Hypersensitivity to Sound (Possibly Hyperacusis) Was Inferred Based on a Positive Answer to the (Written) Question “Do You Have a Hypersensitivity to Sounds (Hyperacusis), Where Normal, Common Sounds Are Unpleasant or Even Painful?” THI: Tinnitus Handicap Inventory.

For the other tests, 16-bit sound waveforms were generated using custom scripts and functions in MATLAB (version R2019a) by a Windows 10 Dell PC equipped with an NVIDIA GeForce GTX 1050 GPU. Both the PC and the experimenter were located outside the sound booth; the PC's monitor output was cloned to an identical P2319H Dell monitor inside the booth. Stimuli were generated digitally at a sampling rate of 44.1 kHz. Sounds were delivered using an E30 USB Digital to Analog audio converter (sampling rate 44.1 kHz; bit depth 16), an L30 headphone amplifier (both from TOPPING, Guangzhou, China), and HDA 200 audiometric headphones (Sennheiser, Wedemark, Germany). The HDA 200 circumaural headphones are more suitable for frequencies above 8 kHz than the supra-aural TDH-39P headphones (ASLHA, 2005). Sounds were always presented to the two ears at equal level. Tone levels were calibrated using an artificial ear (type 4153) and a sound level meter (type 2250, Brüel & Kjær, Nærum, Denmark) at 17 frequencies between 0.5 and 20 kHz. Levels at intermediate frequencies were obtained by interpolation, and after completion of the experiments, it was verified that the calibrations were valid at the f_tp_ values.

Because of the extra tests performed by the tinnitus participants, the tests were spread over 2 test days, which were less than 1 week apart. The controls did all tests (audiometry, loudness-matching, hearing thresholds, psychometric function) on the same day.

#### Loudness-Matching Test

The pitch of low-frequency pure tones tends to decrease with increasing sound level. This effect is small in normal hearing ears (Verschuure & van Meeteren, 1975), but can be much larger in impaired ears (Burns & Turner, 1986). To avoid this effect on pitch estimates or other possible confounding level effects during the pitch, octave confusion or tonality tests, we asked potential tinnitus participants to adjust the level of 18 external tones at frequencies ranging from 0.8 to 16 kHz (4/oct) so as to match the loudness of their tinnitus. Participants adjusted the level of 1-s tones (0.1-s rise and fall times) using a virtual slider, initially with 10-dB steps, and then with 1-dB steps for fine tuning. Each frequency had its own slider, and participants started with the slider for the lowest frequency. Controls were asked to adjust each of the sliders to give a level that was just audible. This level was increased by 10 dB for following tests to reflect the observation that tinnitus loudness typically corresponds to a level of only 5–10 dB SL at f_tp_; at other frequencies, in the absence of loudness recruitment, higher values may have been more appropriate (Henry, 2016). Initial settings at the different frequencies were at a loudness level corresponding to 50 phon for a normal hearing person (ISO 226:2003; https://github.com/IoSR-Surrey/MatlabToolbox, v2.8). This test was also used to find the hearing limit of the participant, i.e., the highest tone frequency that could be heard at the maximum level of 90 dB SPL.

The tinnitus participants were asked to indicate which of the 18 tones best matched their tinnitus pitch. This was used to get an initial estimate for the later pitch-matching test.

#### Tinnitus Masking Test

The tinnitus participant was asked to adjust the level of the tones that were used in the loudness test to a level up to 90 dB SPL to test whether the tinnitus was masked by any of these tones, starting with the tone frequency among the frequencies used in the loudness-matching test that best matched the tinnitus pitch.

#### Pitch-Matching Test

An adaptive (“recursive”) 2-AFC staircase method was used to estimate the tone frequency that best matched the tinnitus pitch (f_tp_), as this method has good reliability (Henry, 2016; Korth et al., 2020; Penner & Bilger, 1992). Staircase procedures were adapted from the PsychoAcoustics toolbox (Soranzo & Grassi, 2014). During each trial, two successive tones were played and the participant had to indicate the two tone whose frequency best matched their tinnitus pitch by pressing “1” or “2” on the keyboard. Based on pilot experiments, the tone level was 10 dB above the (interpolated) value obtained in the loudness-matching test to ensure good audibility, with a maximum of 90 dB SPL. The duration of each of the tones was 1 s, with a silent period of 0.5 s in between. The tone with the lower frequency was always played first to facilitate which spectral direction to follow when there was still a large discrepancy between pitch and estimates. The two starting frequencies were at the approximate frequency estimate obtained at the end of the loudness-matching test and 0.9 oct above this value, which was chosen to be a relatively easy first trial. During the staircase procedure, if the higher (second) of the two frequencies was chosen, this frequency value was assigned to the first tone of the next pair and the second tone had a frequency that was initially 0.9 oct above that of the first one; the reverse happened if the lower of the two frequencies was chosen. If the choice (first or last of the pair) differed from the previous one, it was deemed a reversal. After a reversal, or if one of the two tones was at the edge of the hearing range, the frequency difference between the two tones was reduced. For the first, second, third and fourth set of four reversals, the reductions were 0.5^0.5, 0.5^0.25, 0.5^0.125, and 0.5^0.125, respectively, meaning that after 16 reversals the spectral distance was 1/16 of the difference for the starting pair (0.9 to ∼0.05 oct). The pitch estimate of the test run was the arithmetic mean of the average frequencies (in oct re 1 kHz) at the midpoints of the last four reversals.

The first test run was for practice. If the pitch estimates for the second and third test run differed by less than 0.4 octave, the arithmetic mean of these two values was taken as the pitch estimate. This estimate was then used for the octave confusion and the tonality tests. After each test run, the participant was asked to rate on a 4-point scale (“very good,” “good,” “mediocre,” or “bad”) how well the final tone matched their tinnitus. If they indicated that they had made a mistake or the last presented tones did not resemble their tinnitus at all, that test run was not taken into account, and an additional test run was performed. If the participant did not give a match at least twice with a difference <0.4 octave over a maximum of five test runs (excluding the practice test run), the pitch-matching test was terminated and the participant excluded. The pitch-matching test was repeated on the second day to check whether the pitch estimate was consistent. On the second day, the participant performed a maximum of three test runs (excluding a practice run). If no pair of successive test runs differed <0.4 oct on the second day, or if the mean pitch estimate on the second day differed >0.4 oct from the mean pitch estimate on the first day, the participant was excluded. The pitch estimate for the second day was used for the adaptive hearing threshold and psychometric function tests.

#### Octave Confusion Test

Individuals with tinnitus may show octave confusions in their pitch estimates (Graham & Newby, 1962; Tyler & Conrad-Armes, 1983). A 2-AFC method of constant stimuli was used to verify that pitch estimates were not off by an octave. The participant was presented with two tones and had to indicate the tone whose frequency best matched their tinnitus pitch. The duration of the tones was 1 s, with 0.5 s in between. The two tones were chosen from a set of three tones: the frequency estimated in the tinnitus matching test, a tone one octave higher, and a tone with one octave lower. If the loudness test indicated that the participant was unable to hear a frequency one octave above f_tp_, which was the case in 16 out of 18 participants, the tones were presented at f_tp_, one half octave lower and one octave lower. Based on pilot experiments, tone levels were set to approximately 10 dB above the estimated tinnitus loudness to ensure adequate audibility. Each comparison was presented five times in a randomized block design for a total of 15 comparisons. In the trials with the f_tp_ tone and either one octave below or above, the participant had to choose the f_tp_ tone at least 60% of the trials to continue.

#### Tonality Test

A 2-AFC method of constant stimuli was used for assessing the tonality of the tinnitus. The participant was presented with two successive sounds and had to choose which one resembled the tinnitus the most. The duration of the sounds was 1 s, with 0.5 s in between. The participant compared a tone with level and frequency obtained from the loudness-matching and pitch-matching tests, respectively, with sounds that included some common aspects found for participants with non-tonal tinnitus (Roberts et al., 2008): either ½-octave wide narrowband noise (“hissing noise”), 1/6-octave wide narrowband noise (“ringing noise”), 3-octave wide Gaussian wideband noise, or 3-octave wide Gaussian white noise plus a tone at f_tp_ at equal power. Sounds were centered at f_tp_ and were restricted to the range 0.1–16 kHz. Each comparison was presented six times. The participant had to choose the tone on at least 60% of the trials for each of the four comparisons to continue.

#### Adaptive Hearing Threshold Test

The adaptive hearing threshold test was used to get an estimate of the hearing threshold at the tinnitus match frequency and 0.6 oct below using an adaptive yes/no staircase method. If a hearing threshold in the audiogram had been obtained at a frequency near f_tp_, the starting level was 20 dB above this threshold; otherwise it was 70 dB SPL. The tone duration was 6 s; the participant had to indicate if they could hear it by pressing a key during the 2-s response window, which was indicated by a change from a black to a white color of a circle on the screen 3.5 s after the start of the tone. To make the transitions to a new level less conspicuous, during the first 2 s of the tone, its amplitude gradually (raised cosine) changed from the amplitude of the previous tone to that of the present tone. The absence of a key press signified that the tone could not be heard. The tone level was decreased after a positive response and increased after no response (“1 up/1 down”). The initial step size was 8 dB. A different outcome than the previous choice was counted as a reversal. After the first three reversals, the step size was decreased to 4, 2, and 1 dB, respectively. The staircase finished after an additional five reversals at a step size of 1 dB. The estimated hearing threshold was calculated as the average tone level at these last five reversals.

The control participants were assigned an f_tp_ using a matching procedure. Each control participant was matched with a tinnitus participant based on hearing loss, hearing range, and age. Average hearing loss was calculated by averaging the thresholds obtained from audiometry at 4 and 8 kHz for the two ears; these thresholds were chosen as they were closest to f_tp_ for most subjects. The five members of the tinnitus group with the smallest difference in average hearing loss to the matched control participant were identified. Out of these five, the one was selected whose hearing limit—the maximum frequency that a person could still hear in the loudness test—was closest to that of the matched control participant. In case of a tie, the member of the tinnitus group who was closest in age was chosen. The control participant was then assigned the f_tp_ of the matched tinnitus participant. Multiple control participants could be matched to the same tinnitus participant.

#### Psychometric Function Test

Staircase procedures are relatively efficient in finding thresholds, but they are less efficient for assessing false alarm rate or assessing the slope of the psychometric function (Kingdom & Prins, 2016). A yes/no method of constant stimuli was therefore used to determine the psychometric function near hearing threshold for both f_tp_ and 0.6 oct below. Tone presentation and response window were the same as for the hearing threshold estimate test. Participants were instructed to indicate to press a key if they could hear the sound when the circle on the screen was white (i.e., during the response window). Responses were recorded for eight different levels: the estimated hearing threshold (rounded downward to a whole dB value), 2, 4, and 8 dB above and below this value and a level that could not be heard (∼-20 dB SPL). One run involved 90 trials, 10 per level, except for the lowest level, which was presented 20 times. The order of the sound levels was randomized within blocks of nine stimuli. Two runs were done for each frequency, with a short break in between runs. The sound levels could be shifted 2 dB up or down for the next run (except for the highest level) to get an overall detection rate closer to 50%. The testing order of the two frequencies was alternated between participants. Circles on the computer screen for this test and the hearing threshold estimate test were made using Psychophysics Toolbox MATLAB functions (http://psychtoolbox.org; version 3.0.16), which permitted a precise measurement of key press response delays.

### Analysis

Analysis was performed using MATLAB (R2019a).

#### Psychometric Function Fitting

The data from the psychometric function test were fitted with a Gumbel function using a Simplex procedure and a Bayesian criterion in the Palamedes toolbox (v1.11.11; (Kingdom & Prins, 2016; Prins & Kingdom, 2018). The Gumbel function (also known as the log-Weibull function) is defined as follows:

(1) $$\psi (x)=\gamma +(1-\gamma -\lambda )\times (1-e^{-10^{\beta (x-\alpha )}})$$

where ψ(x) is the fraction of detected sounds as a function of sound level x, γ the false alarm rate (the lower asymptote of ψ), λ the lapse rate (the higher asymptote of ψ), α the threshold level (the sound level at which a fraction of γ + (1-γ-λ)(1-e^−1^) of the sounds is detected), and β the slope parameter (Kingdom & Prins, 2016). The slope parameter β can be converted to the maximum rate of rise β’ (in ψ/dB) using (Strasburger, 2001):

(2) $$\beta^{\prime }=(1-\gamma -\lambda )\times \beta \times \ln\;10/e$$

where ln is the natural logarithm and e is its base. Since γ and λ were typically small, and did not differ between control and tinnitus participants, we report β instead of β’, which avoids a spurious correlation between β and the other fit parameters.

The highest level was 4 or 6 dB above the second highest and could be considered a “free trial”: a trial at which the stimulus level is so high that a detection error can be assumed to be the result of a lapse (Swanson & Birch, 1992). The lowest level was well below hearing threshold (about −20 dB SPL) and was set to -Inf in the psychometric function fits.

Bayesian priors of the threshold were set to a normal function with mean 45 dB and standard deviation of 30 dB. Bayesian priors of the slope were set to a beta distribution with parameters 0.1 and 1.

Data from the two runs at the same frequency were pooled if they did not differ significantly, as judged from the 95% high-density interval of the Bayesian posterior distribution of the threshold difference between the two sessions.

Violin plots were made using the MATLAB function violinplot (https://github.com/bastibe/Violinplot-Matlab).

#### Simulations

To test how well the fit procedure would be able to detect differences in hearing threshold between the two sessions with the same stimulus frequency, we did a simulation. The simulation assessed how well differences in the midpoint threshold level across runs would be picked up. The midpoint of the generating psychometric function was set to the (arbitrary) reference level of 0 dB for the first run; the y-axis of Figure S4 indicates the value of the midpoint on the second run; the midpoint shifts ranged between 0 and 3 dB. Slopes for the two runs were set to the value on the x-axes of Figure S4. As for the psychometric function runs, for each level in a run, 10 samples were drawn, except for the no sound condition, where this was 20, for a total of 90 samples. A sample was obtained by drawing a number from a uniform random number generator with a range between 0 and 1 (function rand in MATLAB), which was scored as a detection if the number was smaller than the detection probability at the stimulus level obtained from the generating psychometric function, and was otherwise scored as a non-detection. The resulting psychometric curve was fitted to obtain an estimate of the change in fit parameters that resulted from the change in the midpoint, as further detailed in the legend of Figure S4.

#### Statistical Analysis

If the participant demographics and other characteristics (Table 2) were not normally distributed, Wilcoxon signed-rank tests were used. Otherwise independent t-tests were used. Tests for normality were done with the Shapiro-Wilk test (https://nl.mathworks.com/matlabcentral/fileexchange/60147-normality-test-package).

**Table 2.** Demographics and Hearing Properties of Participants Without and With Tinnitus.

	Control	Tinnitus	p-value
N	18	18	
Age (yrs)	57.5 ± 8.1	53.8 ± 11.0	0.44
# Men	16 (88.9%)	17 (94.4%)	0.32
f_tp_ (kHz)	9.2 ± 2.7	8.8 ± 2.5	0.70
Hearing loss (dB HL)	27.6 ± 11.3	36.1 ± 14.5	0.046
Hearing limit (kHz)	11.9 ± 1.9	11.5 ± 2.4	0.47
Hearing limit- f_tp_ (oct)	0.43 ± 0.54	0.43 ± 0.53	0.99

Where Applicable, Values Are Shown as Mean±SD. Pitch for the Control Group Is the Assigned Pitch Estimate Based on the Matching Procedure (See Methods). Hearing Loss Was Averaged Over Thresholds Obtained From Audiometry at 4 and 8 kHz for the Two Ears. Hearing Limit Is the Highest Frequency That Could Be Heard in the Loudness-Matching Test. Pitch for the Control Group Is the Average of the Assigned Pitch Estimates Based on a Matching Procedure (Methods). P-Values Were Obtained by Independent-Sample t-Tests or Wilcoxon Signed-Rank Tests, Except Sex Difference, Which Was Obtained From a Χ²-Test.

A multivariate analysis of variance (MANOVA) (one-way; MATLAB function manova1) was used to test whether the means of the slopes (β) and false alarm rates (γ) obtained from the psychometric function fits at the two frequencies differed between participants with and without tinnitus.

To test whether the slope of the psychometric function differed between 0.6 oct below f_tp_ (“low frequency”) and f_tp_ (“high frequency”) for either tinnitus or control participants, we tested whether the posterior estimate of the difference in slope differed significantly from 0.

A mixed-effects ANOVA with tinnitus or control as between-subjects factor (two levels) and frequency (low or high, two levels) and level (eight levels) as within-subject factors was performed to test whether response delays differed between tinnitus and control participants. Only participants with at least one positive response at all frequencies and sound levels were included (n = 6 out of 18 participants for both tinnitus and control). Including all participants would introduce a bias, as participants who responded in trials with low levels or silence were relatively slow responders compared to the participants that only showed a positive response at the higher levels.

Data (anonymized) and software for analysis will be made available upon reasonable request. Software for stimulus generation and testing will be posted to a server that is accessible without restrictions (e.g., GitHub) upon acceptance of the paper.

## Results

### Selection of Participants

Figure 1 summarizes the selection of participants, illustrating that only a small fraction of interested, potential participants with tinnitus turned out to be eligible. For most potential participants, this was already clear from the questionnaire they filled out before visiting the test location. For the potential participants with tinnitus who were tested, the main reasons for not meeting the selection criteria were hearing problems and inability to identify f_tp_ consistently.

A total of 18 participants with tinnitus met all inclusion criteria and none of the exclusion criteria. Their average Tinnitus Handicap Inventory score was 40.1 ± 20.1 (range 14–76). In the octave confusion test, they scored at least 80% on one or both of the comparisons. They scored at least 66% on all four tests in the tonality test. The results of their pitch-matching test are summarized in Figure S1. They typically rated the resemblance of the tone they matched with their tinnitus as good. The average resemblance over the 18 participants during the 2 test days was 3.1 ± 0.4 (mean ± SD), where 4 = very good, 3 = good, 2 = mediocre and 1 = poor.

Each control participant was matched to a tinnitus participant to obtain a test frequency for the psychometric function, as detailed in the Methods. Table 2 summarizes the demographics and other relevant characteristics of participants with and without tinnitus. There was on average an 8.5 dB difference in the average hearing loss at 4–8 kHz, as assessed by clinical audiometry. However, this difference was only marginally significant (p = 0.046, Mann–Whitney U-test, not corrected for multiple testing), and, more importantly, thresholds obtained from the psychometric curve tests did not differ significantly between tinnitus and control participants both at f_tp_ (63.6 ± 19.7 vs. 61.2 ± 16.9 dB SPL; p = 0.70, t-test) and at 0.6 oct below f_tp_ (49.9 ± 16.9 vs. 39.6 ± 15.6 dB SPL; p = 0.065, t-test).

Table 2 shows that the difference between the hearing limit, the highest tone frequency that could still be heard, and f_tp_ was only ∼0.4 oct on average. In many cases, especially for older participants, f_tp_ was close to the hearing limit (Figure S2).

### No Difference in Psychometric Function Threshold, Slope and False Alarm Rate Between Participants With and Without Tinnitus

**Figure 2.**
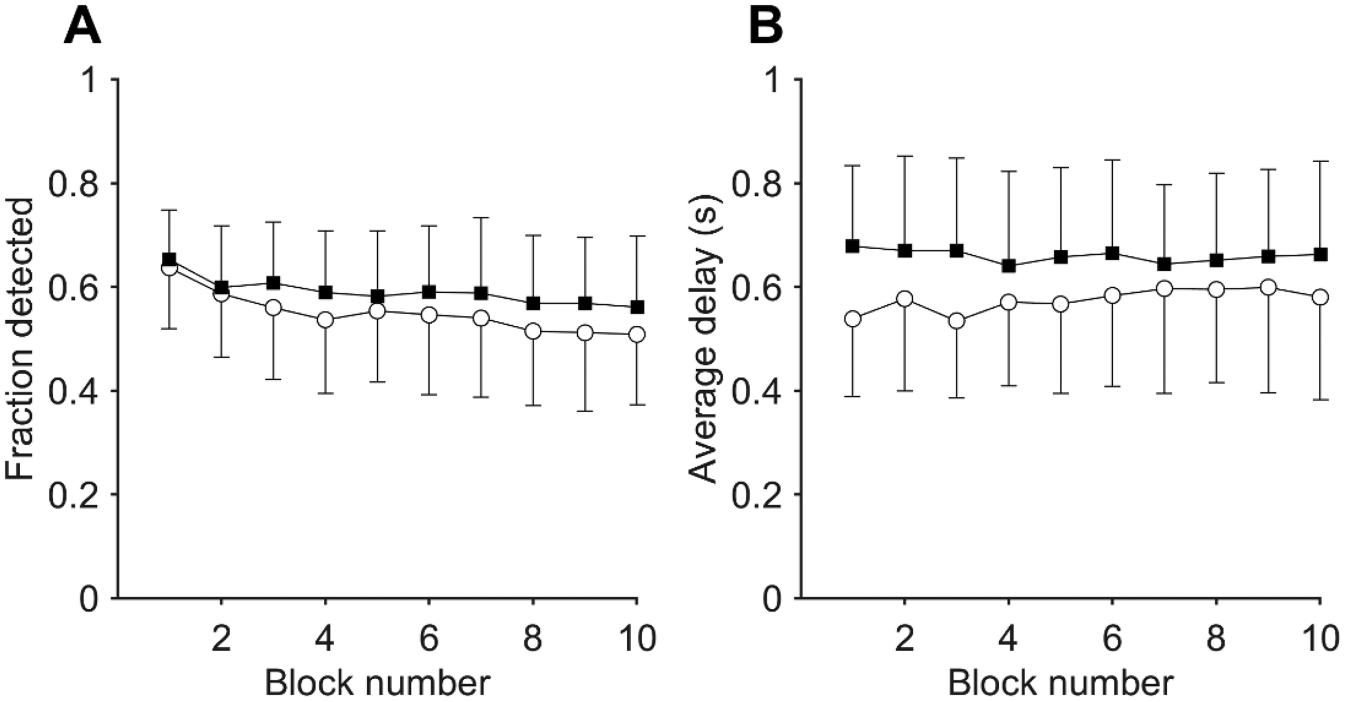
Responses and Response Delays During the Psychometric Test. (A) Block-Averaged Fraction of Detected Stimuli in Participants With Tinnitus (Closed Squares; n=18) and Control Participants (Open Circles; n=18). Each Block of Nine Stimuli Contained the (Randomized) Eight Different Stimulus Levels, With the Silence Presented Twice. Error Bars Denote S.D. (B) As (A), but Showing Block-Averaged Response Delays.

The participants performed a psychometric function test both at f_tp_ and at a frequency 0.6 oct below. Participants generally remained concentrated during a run, as judged from the relatively constant average hit rate and response delay over the different stimulus blocks (Figure 2) and the overall low rate of missing a detection at the highest level (0.008 ± 0.035, n = 36, averaged over the four sessions; only three participants missed one or more detections at the highest level). Participants generally had similar performance during the two runs.

**Figure 3.**
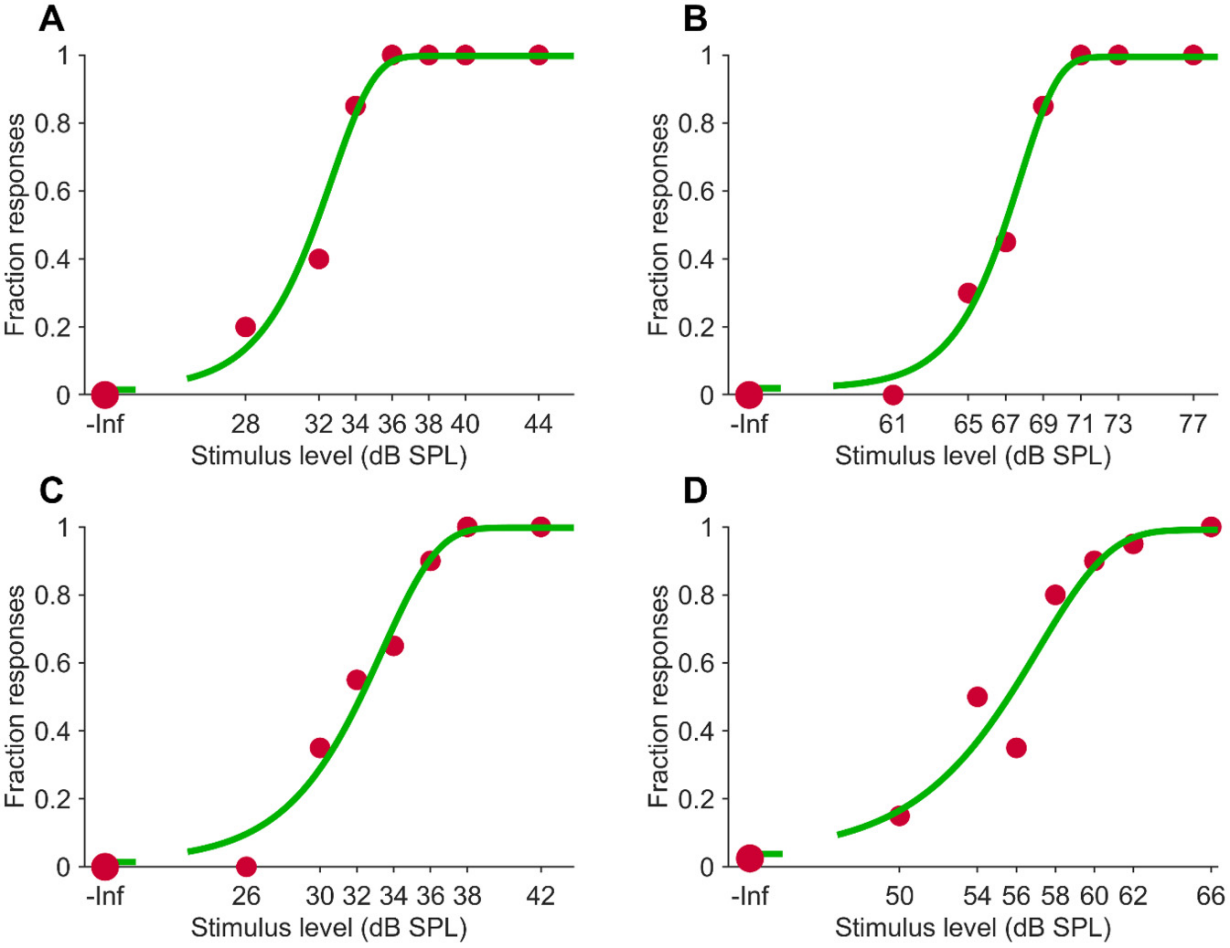
Psychometric Function Examples. Red Filled Circles Show Positive Responses. Symbol Size Is Proportional to Number of Repeats (20 vs. 40). Green Line Is Fit With Gumbel Psychometric Function. (A) Control Participant, 8.1 kHz, Matched Pitch, Threshold 32.8 dB SPL, Slope 0.19, False Alarm Rate 0.02, Lapse Rate 0.003. (B) Control Participant, 12.3 kHz, 0.6 Oct Below Matched Pitch, Threshold 67.5 dB SPL, Slope 0.21, False Alarm Rate 0.02, Lapse Rate 0.006. (C) Tinnitus Participant, 4.3 kHz, f_tp_, Threshold 33.6 dB SPL, Slope 0.14, False Alarm Rate 0.02, Lapse Rate 0.002. (D) Tinnitus Participant, 6.5 kHz, 0.6 Oct Below f_tp_, Threshold 56.8 dB SPL, Slope 0.12, False Alarm Rate 0.04, Lapse Rate 0.009.

The data for the two runs were fitted with a psychometric function (Gumbel) using a Bayesian criterion, as described in the Methods. Fits were generally quite adequate, as illustrated by the observation that thresholds could be estimated with an average error of <0.7 dB (n = 72). The two runs at the same sound frequency were combined if the two threshold estimates did not differ significantly, as judged by the difference in the posterior threshold distributions. For 10 out of the 72 cases, the difference was significant, and for these cases, the run that gave the most precise estimate of the threshold was chosen. Figure 3 shows examples of combined runs and the fits for the two frequencies for a tinnitus and a control participant.

**Figure 4.**
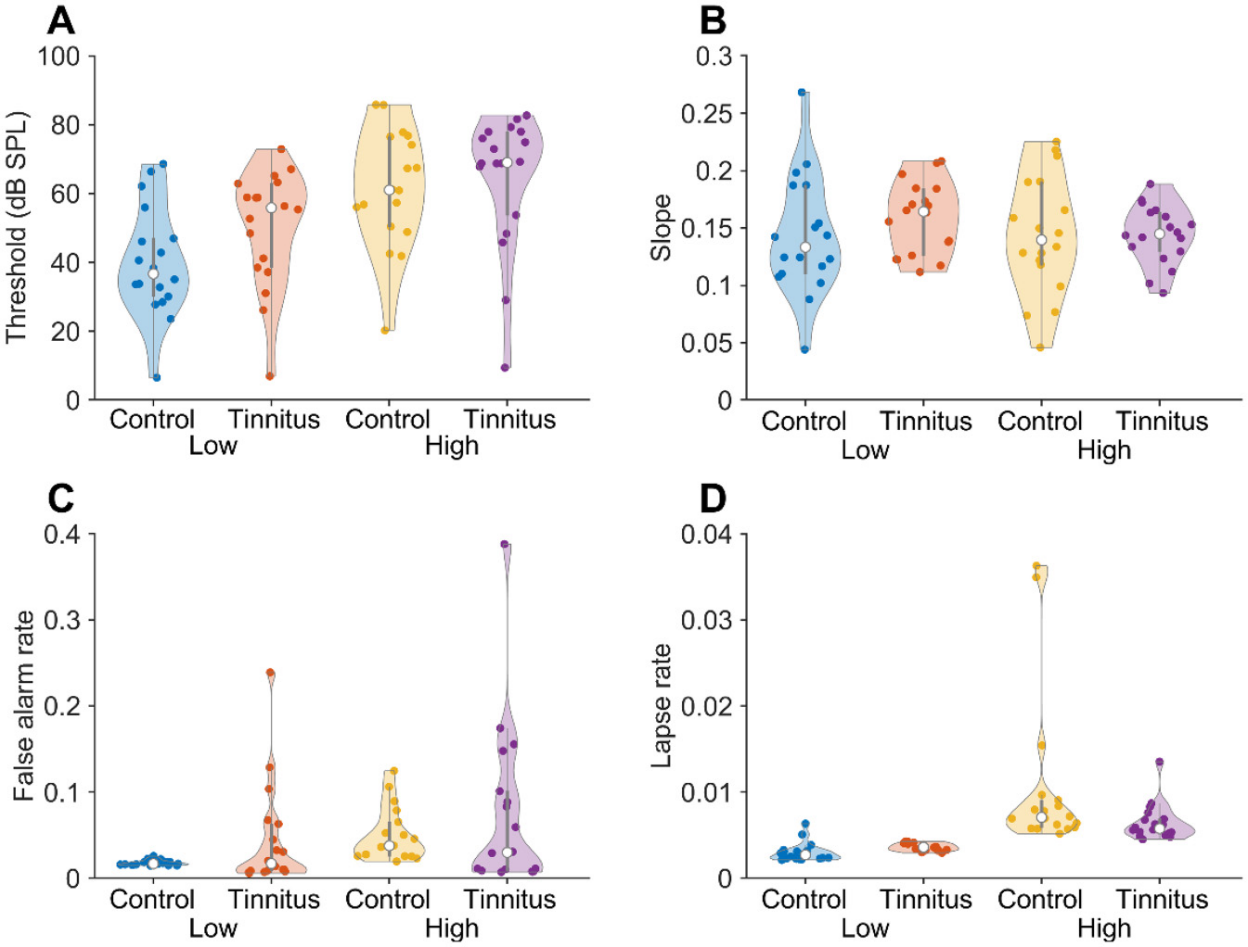
Summary of Fit Results. Violin (Swarm) Plots of Fit Parameters. The Open Circle Denotes the Median; the Thicker Vertical Lines the Boundaries of the First and Third Quartiles; Colored Circles Show the Individual Data Points. (A) Threshold Fit Parameters. (B) Slopes. (C) False Alarm Rates. (D) Lapse Rates.

The fit results are summarized in Table 3 and Figure 4. We used a MANOVA to test whether the means of the slopes and false alarm rates obtained from the psychometric function fits at the two frequencies differed between participants with and without tinnitus. No significant difference was found (p = 0.28). In agreement with this, the posterior estimate of the difference in slope at 0.6 oct below f_tp_ and f_tp_ did not differ significantly from 0 (95% high-density interval −0.083–0.169; mean difference 0.038). The same held true for the control group (95% high-density interval −0.118–0.122; mean difference 0.001). The lack of difference in the slope for the two tested frequencies for the control group is in general agreement with Watson et al. (1972), who found no dependence of slope on frequency in the range 1–4 kHz, whereas Zhou and Green (1995) found a 25% increase in the slope between 1 and 15 kHz.

**Table 3.** Summary of Fit Results.

	Control-low	Control-high	Tinnitus-low	Tinnitus-high
Threshold	40.0 ± 15.9	61.5 ± 17.0	50.1 ± 16.9	64.1 ± 19.7
Slope (β)	0.14 ± 0.052	0.14 ± 0.051	0.16 ± 0.031	0.14 ± 0.025
False alarm rate	0.018 ± 0.0031	0.050 ± 0.031	0.045 ± 0.060	0.074 ± 0.097
Lapse rate	0.0030 ± 0.0011	0.011 ± 0.0094	0.0036 ± 0.0004	0.0065 ± 0.0021

Values Are Given ± S.D. For All Values n = 18. In the Column Titles, Low and High Refer to the Tone Frequency: 0.6 Oct Below and at f_tp_, Respectively.

### Response Delays

If tinnitus induced uncertainty about the detection of external sounds, response delays would be expected to be relatively large for the tinnitus participants at f_tp_, especially in silence, compared to the delays at 0.6 oct below f_tp_, or compared to the control participants at f_tp_. However, there were no obvious differences in the effects of stimulus frequency or stimulus level on response delay between participants with and without tinnitus (Figure S3). Average delays were 0.66 ± 0.12 and 0.67 ± 0.13 ms (mean ± s.d.) for the tinnitus participants at 0.6 oct below f_tp_ and f_tp_, respectively, and 0.56 ± 0.12 and 0.58 ± 0.12 for the control participants at 0.6 oct below f_tp_ and f_tp_, respectively. A mixed-effects ANOVA was performed to test whether response delays differed as a function of frequency and level between tinnitus and control participants. There was no significant main effect of tinnitus vs. control (F(1,10) = 3.93; p = 0.076), or frequency (F(1,10) = 0.8, p = 0.39). Even though, as expected, response delays were shorter for louder stimuli, the main effect of level did not reach significance (F7,70) = 2.1, p = 0.054). There were also no significant interactions between frequency, level and tinnitus. These results therefore do not support the idea that tinnitus induced uncertainty about the presence of an external sound. However, there are some limitations to this conclusion. The participants were not instructed to respond as fast as possible. Also, mostly due to the low response rate in silence, the number of included participants was low, only 6 (of 18) tinnitus and 6 (of 18) control participants.

### Simulations

The lack of a significant difference in fit parameters could be due to inaccuracies introduced by pooling the data for the two sessions in which the same tone frequency was tested. Especially at high frequencies, thresholds are sensitive to the positioning of the headphone (Paquier et al., 2016; Watson et al., 1972; Zhou & Green, 1995), which were taken off between sessions. However, simulations showed that the data fitting procedure should be able to discern even small differences in threshold, and that the observed differences in threshold would have only a minor effect on the estimated slope in the combined data (Figure S4). Thresholds could be accurately estimated for slopes between 0.05 and 0.5 (Figure S4A), i.e., over the range of observed slopes. Variance in the fitted threshold parameter was low in that range (Figure S4B). At a steep slope, threshold differences became significant at 1.5 dB or more. At shallow slopes differences of 3 dB or more were needed to get significant threshold differences (Figure S4C). Importantly, the simulations showed that differences in the underlying thresholds up to 1.5–2 dB would not introduce a substantial systematic bias in the fitted slope values (Figure S4D).

## Discussion

### Main Findings

Our results indicate that (tonal) tinnitus does not interfere with the detection of soft sounds with a frequency that best matches its pitch. We did not find a significant difference in the false alarm rate, the slope of the psychometric function or the response delay at f_tp_ compared to a lower frequency in tinnitus participants, or compared to control participants. Even though by selecting participants with symmetric, tonal tinnitus we maximized the likelihood for confusion with an external sound, our results suggest that individuals with tinnitus apparently discriminate well between their tinnitus and external sounds.

### Matching Tinnitus with an External Tone

To maximize the likelihood of participants confusing their tinnitus and an external sound, we selected participants with tonal tinnitus, who could match its pitch consistently. Many potential participants were not able to do this (Figure 1), in agreement with previous results showing that individuals with tinnitus generally find it difficult to match the pitch of their tinnitus consistently (reviewed in Henry (2016). This may be partly related to hearing problems that are often associated with the presence of tinnitus. For example, if the matching frequency falls in a cochlear “dead” (non-functional) region, the matching tone may not have a clear pitch (Huss & Moore, 2005). Despite our efforts to select only individuals with tonal tinnitus with relatively good matching abilities, participants were apparently not sufficiently confused to significantly affect false alarm rate or the slope of the psychometric function at f_tp_. Individuals with tinnitus do have to distinguish between their tinnitus and external sounds in everyday life, so they may well have become experts at this. It may also have been the case that the external tone simply did not match their tinnitus percept very well. Indeed, the resemblance of the matched tones to the tinnitus was typically rated as “good,” but not as “excellent.” Interestingly, the majority of the tinnitus participants gave a pitch estimate that was within 0.4 oct of the highest tone frequency that could be heard (Figure S2). This is noteworthy for two reasons. First, 0.4 oct is also the maximum range that was accepted for being able to match the pitch consistently. This means that we may have selected individuals who consistently estimated their pitch to be close to their hearing limit. Second, participants obviously could not give a match above their hearing limit. Individuals with preserved high-frequency hearing up to 8 kHz tend to report a relatively high f_tp_ (>8 kHz) for their tinnitus (Pan et al., 2009). The participant in our study were often older participants with coexisting hearing loss, raising the possibility that due to their hearing loss the tone that best matched their pitch had become inaudible, and that they reported the audible tone that came closest. The relationship between tinnitus pitch and hearing loss is not simple (Huss & Moore, 2005; König et al., 2006; Moore et al., 2010; Norena et al., 2002; Pan et al., 2009; Schecklmann et al., 2012; Sereda et al., 2011; Shekhawat et al., 2014; Tyler & Conrad-Armes, 1984; Yakunina & Nam, 2021); most studies, including this one, are cross-sectional and suffer from small sample sizes. Also, most studies have only considered frequencies up to 8 kHz. A (longitudinal) population study could permit testing whether the f_tp_ of someone with presbycusis shifts to lower frequencies along with the shift in the highest frequency that can still be heard.

### Comparison with Earlier Results

The psychometric functions for participants with tinnitus had similar slopes at f_tp_ and below. This is in agreement with the observation that tinnitus behaves differently from an external tone matched to the tinnitus pitch in masking experiments (Penner & Klafter, 1992). However, our results differ from those of Penner (1986), who found shallower slopes of the psychometric functions at f_tp_ than at lower or higher frequencies for 6 of 7 participants with tonal tinnitus. There are a number of possible explanations for this discrepancy. First, there are differences in test design. Penner (1986) used a two-interval forced-choice method to estimate the detection threshold for external tones; we used a yes/no task, which allowed us to measure the false alarm rate, and we used long-duration tones. Long-duration tones have the advantage of resembling tinnitus, which is also long-lasting, and masking of the tinnitus by the tone may make it more difficult to tell which is which. Long-duration tones have the disadvantage that intense tones may induce adaptation. Indeed, the slope of the psychometric function for both tinnitus and control groups was on average shallower in our study than in Penner (1986), which may have been due to adaptation. Another difference is that in Penner (1986), 5 of 7 participants had monaural tinnitus, whereas we selected participants with bilateral tinnitus. In Penner (1986), the clearest slope differences were found for a frequency that was higher than f_tp_, whereas we could only test a frequency below f_tp_, partly owing to the substantially higher f_tp_ values in our study (5.4 ± 3.4 vs. 8.8 ± 2.5 kHz; p = 0.039).

The lack of evidence that the participants with tinnitus confused their tinnitus with external tones is in agreement with Feldmann (1971), who did a tinnitus masking study with a large sample of tinnitus participants, and found that almost everyone was able to clearly distinguish between their subjective tinnitus and the external masker sound.

### Tinnitus Test

The lack of a difference between control and tinnitus participants in the slope of the psychometric function in our study is in line with the observation that it has been difficult to find a hearing test that robustly and objectively discriminates between tinnitus and control participants at an individual level (Langers et al., 2012; Long et al., 2023; Nieschalk et al., 1998; Omidvar et al., 2018; Ravirose et al., 2019; Reisinger et al., 2024; Tan et al., 2013; Zeng et al., 2020). Moreover, some of the results have been contradictory. For example, whereas some studies found decreased speech-in-noise or competing speech perception (Gilles et al., 2016; Liu et al., 2020), other studies found that speech-in-noise perception was equal to (Oosterloo et al., 2020) or even better (Zeng et al., 2020) for individuals with tinnitus than for controls.

### How Many Neurons Involved?

Whereas tinnitus is highly prevalent and presents a significant burden to many individuals, many of its properties are elusive. The difficulty in finding a hearing test in which individuals with tinnitus consistently perform differently than controls, the difficulty individuals with tinnitus have in describing their tinnitus, the finding that almost any audible sound can mask the tinnitus effectively in some individuals with tinnitus, the long periods that the tinnitus may disappear following prolonged exposure to sounds (“residual inhibition”): all these well-known properties of tinnitus attest to its elusive qualities. What might account for this? Fowler ascribed the interference of tinnitus with normal hearing to the “busy wire” effect: the neurons that are busy with the tinnitus cannot be used for the detection of external sounds (Fowler, 1941). Our results suggest that it is difficult to demonstrate that the lines are indeed busy perhaps indicates that tinnitus occupies only a small portion of the available bandwidth. The question what neural activity is minimally needed to get a tinnitus percept has not received much attention in the literature. For conscious sound perception, many neurons are involved. Close to the auditory detection threshold, both undetected and consciously detected sound stimuli show activity in early auditory regions, including thalamus and primary auditory cortex, but with differences in activity between detected and non-detected stimuli (Christison-Lagay et al., 2025). Responses to the detected but not to the undetected auditory stimuli subsequently propagate outward to higher order regions, which may include frontal regions (Blumenfeld, 2023; Christison-Lagay et al., 2025; Hillyard et al., 1971; Mashour et al., 2020; Panagiotaropoulos, 2024; Sanchez et al., 2020; van Vugt et al., 2018). However, these studies indicate that large number of neurons is involved in the conscious experience, but not about the minimum number of neurons that is needed to instigate a sensory percept. Animal research has shown that this number is surprisingly small; only tens of cortical neurons are needed (Dalgleish et al., 2020; Marshel et al., 2019; Tanke et al., 2018). Whereas these numbers are not yet known for the auditory periphery, there is no reason to assume that they would be much larger, as the total number of auditory neurons is much smaller in the auditory periphery. This finding argues that a small number of neurons with abnormal firing would be sufficient to get a tinnitus percept. If these neurons are close to each other in a tonotopically organized region, the percept would likely be tonal. However, even adjacent neurons may already have quite heterogeneous properties in the auditory periphery (Barnstedt et al., 2015; Geis et al., 2011; Wong & Borst, 2019), suggesting that a tonal percept becomes unlikely for large ensembles of hyperactive neurons. We therefore conclude that our results are compatible with the idea that a small number of nearby cells in a tonotopically organized region can cause tonal tinnitus. If correct, this means that despite the substantial progress that has been made in recent years in identifying the underlying mechanisms of tinnitus (Baguley et al., 2013; Henton & Tzounopoulos, 2021; Shore & Wu, 2019), finding these cells among the ubiquitous spontaneous activity in the brain (Eggermont, 2015; Meyer-Baese et al., 2022) and investigating how they cause tinnitus is still a formidable challenge. A further challenge is to understand how the activity of these cells can be so easily masked in some cases. Their signals would be too small to be detected by fMRI, EEG, or MEG, suggesting that animal experiments might be the path forward. However, it is difficult to determine whether an animal has tinnitus, where both reflexive methods and methods based on operant conditioning rely to a large extent on confusion of internal and external sounds (Galazyuk & Brozoski, 2020). Our study indicates that this may not be a good basis for a tinnitus model in animals. As challenging as this may be, the large disease burden and the current paucity of treatment options warrants a concerted effort to elucidate tinnitus mechanisms.

## Supplemental Material

sj-docx-1-tia-10.1177_23312165251376382 - Supplemental material for Tonal Tinnitus Does Not Interfere with Tone Detection at the Tinnitus Pitch-Matched Frequency Supplemental material, sj-docx-1-tia-10.1177_23312165251376382 for Tonal Tinnitus Does Not Interfere with Tone Detection at the Tinnitus Pitch-Matched Frequency by J Gerard G Borst and André Goedegebure in Trends in Hearing
