# Supplementary material for: Tonal Tinnitus Does Not Interfere with Tone Detection at the Tinnitus Pitch-Matched Frequency
Source: Trends Hear. 2025 Sep 8;29:23312165251376382. doi: 10.1177/23312165251376382 (PMC12618831; doi:10.1177/23312165251376382)
Supplement: sj-docx-1-tia-10.1177_23312165251376382 - Supplemental material for Tonal Tinnitus Does Not Interfere with Tone Detection at the Tinnitus Pitch-Matched Frequency [file sj-docx-1-tia-10.1177_23312165251376382.docx]

**Supplementary figures**


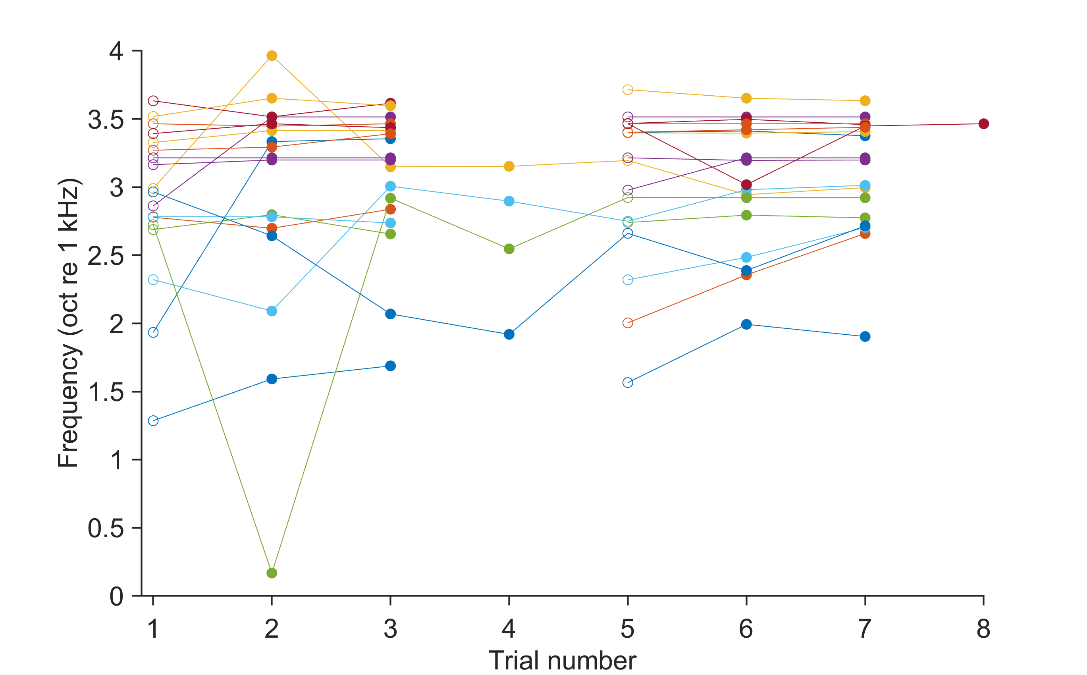


**Fig. S1 Results of the pitch matching test for the participants.**

Practice trials are shown as open circles, test trials as closed. The first two trials after the practice trial on the first test day were within 0.4 oct in 14 of 18 participants (mean absolute difference 0.05 oct). For the other four, the next extra trial (trial 4) was within 0.4 oct of the preceding one (mean absolute difference 0.13 oct). The difference between the two trials after the practice trial on the 2nd day (trial 6 and 7 in the graph) had to be within 0.4 oct, which was the case in 17 of the 18 participants (mean absolute difference 0.07 oct). In addition, the average of these two trials had to be within 0.4 oct of the 1st day pitch estimate, which was the case for all 18 participants (mean absolute difference 0.12 oct, n = 18)


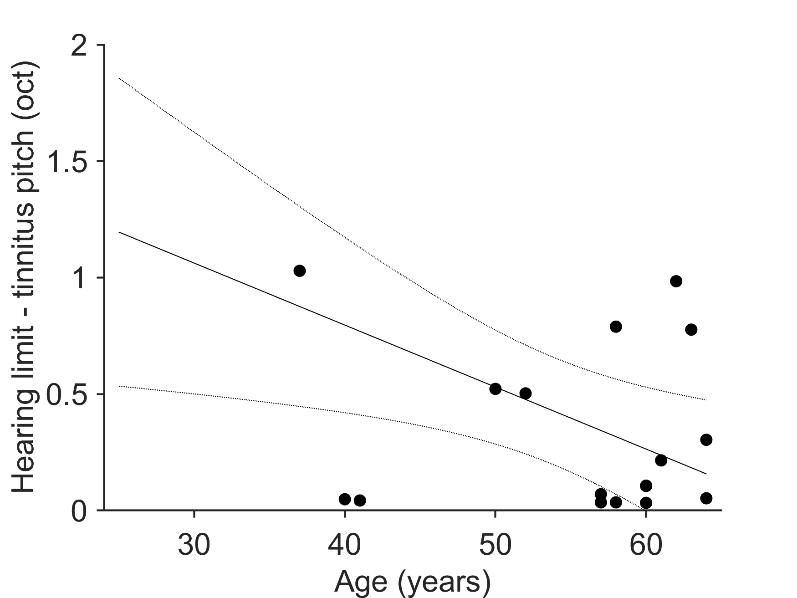


**Fig. S2 Age dependence of the difference in hearing limit and tinnitus pitch.**

Filled circles are the tinnitus participants (n = 18). Solid line is the regression line (r = -0.55; p = 0.018). Broken lines are the 95% confidence bounds


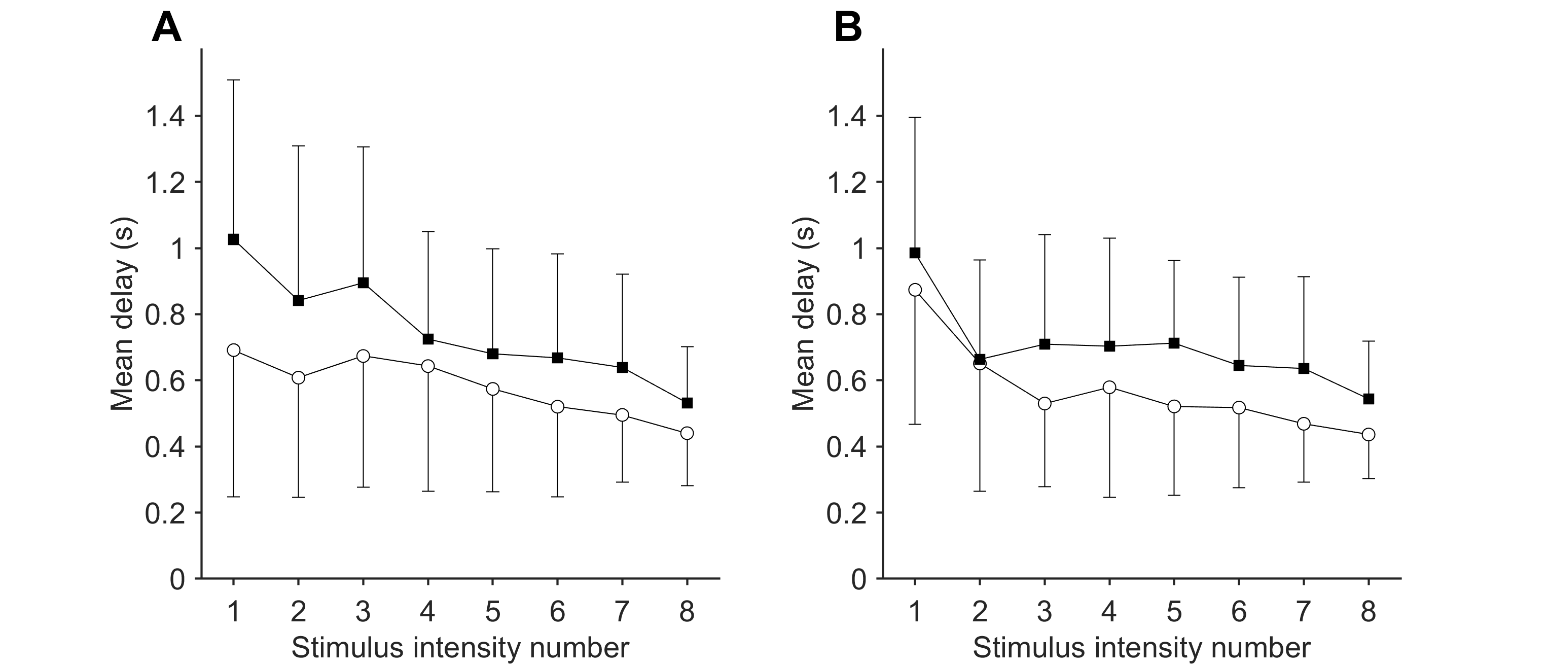


**Fig. S3 Response delays as a function of sound frequency and level.**

A. Response delays at f_tp_ in participants with tinnitus (closed squares; n = 6) and control participants (open circles; n = 6). The horizontal axis denotes sound level, with ‘1’ signifying no sound and ‘8’ the sound with the highest level presented within a session. B. As A, but for test frequency 0.6 oct below matched pitch. Error bars denote s.d.


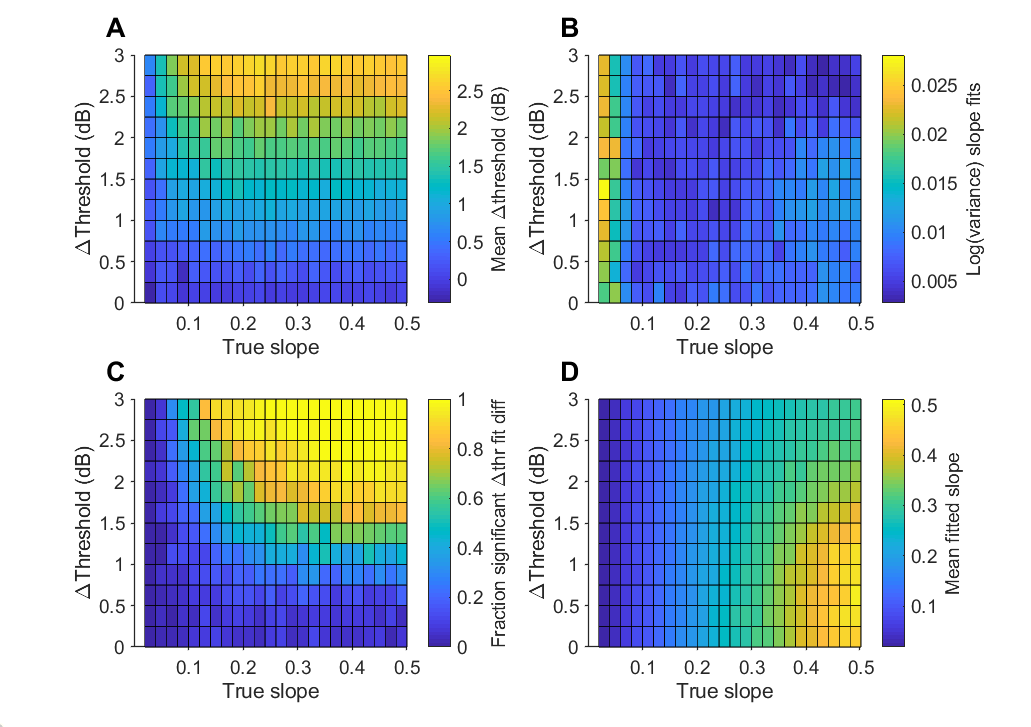


**Fig. S4 Simulation of the detectability of threshold changes between sessions.**

A. Difference in fitted threshold as a function of slope (horizontal axis) and the difference in threshold (vertical axis) in the underlying psychometric function. For slopes >0.05, the observed difference in threshold generally matches the ‘true’ difference in threshold (cf. colour of data point and colour of y-coordinate from colour scale). B. As A, but showing the variance in the fitted threshold parameter. C. As A, but showing the fraction significant differences in threshold. Significant differences were assessed based on the Bayesian posterior estimates of the threshold parameter in both runs. D. As A, but showing the average fitted slope parameter. For large differences in threshold (>2 dB) steep slopes will be underestimated. Each pixel is the average of 100 repetitions
